# An economic analysis of varicella immunization in the Singapore military

**DOI:** 10.1186/s40779-016-0070-9

**Published:** 2016-02-03

**Authors:** Jake J. K. Goh, Marc Ho, W. M. Koh, Vernon J. Lee

**Affiliations:** Specialist Medicine Centre, AFPN 0047, 27 Medical Drive #08-01, Singapore, 117510 Singapore; National University of Singapore, MD3, 16 Medical Drive, Singapore, 117597 Singapore

**Keywords:** Varicella, Vaccination, Military, Singapore, Economic analysis

## Abstract

**Background:**

Varicella outbreaks occur frequently in closed environments such as those of militaries. This paper compares the economic outcomes of varicella vaccination in enlisted servicemen without prior reported varicella infection or vaccination.

**Methods:**

We analyzed the economic outcomes of a varicella vaccination program on all enlisted servicemen without prior reported varicella infection or vaccination in the Singapore Armed Forces (SAF) between December 11, 2010 – December 20, 2013, compared with the previous program of varicella vaccination only for selected personnel between December 1, 2007 – December 10, 2010.

**Results:**

In the at-risk population of all active SAF servicemen, the program of varicella vaccination for all servicemen without prior reported varicella infection or vaccination upon enlistment would save 72.0 work days per 1000 (95 % CI: 61.2 - 82.9), valued at SG$6,544 per 1000 (95 % CI: 6,524 - 6,564), i.e., costing SG$91.5 per work day saved (95 % CI: 78.7 - 107.3). This also results in a reduction of 2.7 varicella cases per 1000 and 5.43 outbreaks per 10000, or a total savings of SG$1,695 per 1000 (95 % CI: −2,730 - 6,834), taking into account the cost of work days lost over a three-year period, compared with the previous regime of vaccinations only for selected individuals.

**Conclusion:**

The varicella vaccination strategy targeting all enlisted servicemen without prior reported varicella infection or vaccination is able to prevent varicella infections and outbreaks, thus reducing absenteeism and days lost.

## Background

Varicella zoster virus (VZV), the etiological agent of chickenpox [1], can cause clinical illness lasting up to 2 weeks and results in substantial morbidity and absenteeism from work. In addition, because the infectious period begins when respiratory symptoms occur [2], the public health management of outbreaks is difficult in densely populated military camps. However, studies conducted in the United States [3–5] and Israel [6] have shown that the vaccination of newly recruited military servicemen susceptible to varicella was a cost-effective measure that reduces the incidence of chickenpox and hospitalization. So far, there have been no similar studies conducted for militaries based in the tropics, and this paper serves to fill this gap in the literature.

Although a live attenuated VZV vaccine is available and is included in the recommended vaccination schedule for children in many countries [7], it is not compulsory to vaccinate against varicella in Singapore [8], a city-state in tropical South-East Asia with a population of approximately 5.47 million as of 2014. Thus, many among the approximately 20,000 young, able-bodied male Singaporeans who are enlisted into the military each year are susceptible to chickenpox during military training due to the close living quarters and training environments.

The varicella vaccine is generally effective, safe, and provides long-lasting immunity. Clinical trials demonstrated that the vaccine is 70 % to 90 % effective for preventing varicella, more than 95 % effective for preventing severe cases, and confers protection ranging from at least 11 to 20 years [9, 10]. The long-term duration of protection from the varicella vaccine is unknown, but there are now persons vaccinated more than thirty years ago with no evidence of waning immunity, while others have become vulnerable in as few as six years [11]. The vaccine is considered safe, as only approximately 5 % of individuals develop mild symptoms after vaccination, and serious side effects from the varicella vaccine are very rare. There was a case of the varicella-associated death of a vaccinated child with leukemia in California 2012, though this was likely the result of profound immunosuppression in part from chemotherapy and corticosteroid treatment [12].

Although chickenpox is unlikely to cause severe illness, the 1- to 2-week long recovery period disrupts training schedules and puts a strain on the operational readiness of the military. Furthermore, outbreaks are time consuming and potentially expensive, as man-days are wasted in contact tracing, the vaccination of susceptible trainees and in performing daily screening. To reduce these outbreaks, a varicella vaccination program was instituted on December 11, 2010 for all enlisted personnel without a reported history of varicella infection or vaccination. This was in line with a previous study showing that the recalled history of varicella infection was a good predictor of serological immunity [13].

## Methods

### Study design

We compared the outcomes and cost benefits of a comprehensive varicella vaccination program for enlisted servicemen for a 3-year period from December 11, 2010 to December 20, 2013 in comparison to the preceding 3-year period from December 1, 2007 to December 10, 2010. Since December 11, 2010, susceptible enlisted servicemen (24 % of the military) have been administered the varicella vaccination by trained medics within one week of their enlistment. Under the previous program, only certain servicemen considered to be more susceptible to varicella infection (such as healthcare workers and naval personnel) were vaccinated for varicella. The decision made to determine “high-risk groups” (10 % of the military) was for operational reasons, such that their potential to spread the virus or experience downtime due to varicella infection was considered unacceptable by the military leadership; thus, vaccination was mandated for this group.

### Measures

This study compares the cost benefits of varicella vaccination upon enlistment for all enlisted SAF servicemen without prior vaccination or infection compared to having varicella vaccination for only selected servicemen from the perspective of policy-makers in the SAF. Primarily, the costs to the SAF (e.g., vaccination and medications) were compared with the benefits (e.g., the number of varicella cases averted and work days saved) within the at-risk population of all active military personnel via a sensitivity analysis. Varicella cases are clinically diagnosed, and cases typically present with respiratory symptoms, fever, maculopapular rashes and/or contact history. Recorded clinical cases of varicella were diagnosed by SAF Medical Officers (MOs), who are qualified medical practitioners in Singapore with a basic medical degree and are fully registered with the Singapore Medical Council, having also graduated from the SAF’s Medical Officer Cadet Course. Laboratory confirmation and random testing for varicella is not routinely conducted and thus the system will not be able to pick up asymptomatic and subclinical cases. This means that this paper might underestimate the incidence of varicella, but equally so for the two time periods.

### Data collection

All clinic entries with “varicella” or “chickenpox” as part of the diagnosis were selected from the SAF's electronic medical records (which uses ICD-9 codes for diagnoses). We collected information including sex, race, date of birth, medical leave duration and prescribed medication. Microsoft Excel and RStudio [14] were used for the data analysis.

As all of these patients were seen by SAF MOs, and their clinical encounters were recorded into electronic medical records, including the subsequent management of the patient by the MO. Most patients were prescribed with symptomatic medications, primarily paracetamol, NSAIDs, calamine lotion and anti-histamines, while a handful were prescribed antivirals. The estimated cost of each outpatient consultation including symptomatic medications is estimated to be SG$34.50 (all analyses are in SG$, using the 2014 exchange rate at SG$1.28 to US$1) [15]. For this analysis to be robust, we allow the cost of medication to vary uniformly within 20 % of this value.

Appropriate direct and indirect economic costs were included in the analysis (Table 1). These costs include comparing the number of outpatient varicella cases, the total number of outpatient costs, the number of days lost, the number of outbreaks and the total number of vaccines administered during the 2 time periods.

**Table 1:** Economic data

Item	Data
Cost per outpatient consultation (including medications) (SG$)	34.50
Estimated days lost per case due to sick leave/disease	6.57 days (2.76 s.d.)
Cost of one lost day (SG$)	174.00
Cost of outpatient treatment (SG$)	34.50 (27.6-41.4)
Cost per vaccine vial (SG$)	30.95
Cost of side effects of vaccine per person (SG$)	174.00
Growth rate of median wages^a^	6.093 %
Man-day lost from a varicella outbreak	100 (80–120)
Side effect rate	5 %
Discount rate^b^	1.237 % annual

^a^Calculated using data from the Ministry of Manpower, Singapore, 2013;

^b^Geometric mean of 5-year SGS Bond Yields from 2007 to 2014. Bond yields are obtained from https://secure.sgs.gov.sg/fdanet/BenchmarkPricesAndYields.aspx

The value of one lost day of work, SG$174 (or SG$3,460 a month), was estimated from the Ministry of Manpower's median gross monthly income from work of full-time employed residents in 2012. Median gross wages experienced a geometric growth rate of 6.093 % from 2007 to 2013 [16]. For this analysis, we allowed income to vary between SG$2,400 to SG$4,400.

Discounting was performed using the Geometric Mean Return of five-year (5Y) Singapore Government Securities (SGS) from 2007 to 2014, which represents the time value of money over the 6-year research period from 2007 to 2013.

The number of man-days lost due to symptomatic varicella cases was calculated from the duration of medical leave granted to each patient. Missing data can occur because military staff can choose to visit external (non-SAF) General Practitioners (GPs) for treatment, but these cases are still captured in the system because medical leave beyond 3 days in duration is endorsed by an SAF MO. However, the system will not record the number of days of leave granted by the external doctor. These cases will appear in the data as varicella patients with 0 to 2 days of medical leave. A subset of 121 of 435 cases fall under this category, and the ‘true amount’ of medical leave is imputed using the distribution of medical leave for the other 314 personnel. The average number of work days lost from varicella after statistical imputation was 6.57 days (2.76 s.d.). This simpler method is chosen over regression imputation because the number of days of medical leave taken does not appear to have any correlation with the demographic variables of the patients.

The administration costs of varicella vaccinations are assumed to be negligible, as the mass vaccination of servicemen upon enlistment for varicella is part of an existing vaccination program for other communicable diseases. Each vaccine vial administered costs SG$30.95, according to data taken from the SAF's vaccine vendor. Approximately 5 % of personnel vaccinated are estimated to suffer from one day lost due to side effects such as fever and rash [17].

An outbreak is defined as 2 or more cases of varicella, including the index case, in the same unit within 3 weeks of the previous case. An outbreak is estimated to cost approximately 100 work days, based on the time and effort required to activate the Preventive Medicine Unit and camp MOs for contact tracing and the daily screening of contact cases and to vaccinate the non-vaccinated servicemen from the index case’s company of soldiers. We allowed the outbreak work day cost to vary between 80 to 120 days in our cost-benefit analysis.

### Sensitivity analysis

**Fig. 1:**
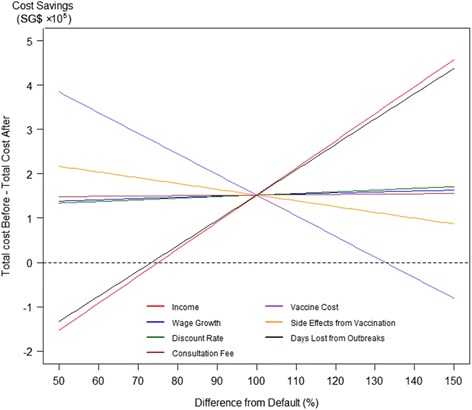
Sensitivity analysis. The diagram shows the effect on the final outcome (Total cost before intervention - Total cost after intervention) when seven distinct variables are shocked −50 % to 150 % of the original value. The intervention is a sound investment if the graph stays above 0 (i.e., the cost before > cost after)

A sensitivity analysis was performed to identify the variables that influence outcome. The cost savings from the varicella intervention was valued at 2014 values in SG$. The default settings were as follows: SG$3400 monthly income in 2012, wage growth of 6.09 %, 1.237 % annual discount rate (0.1025 % per month), SG$34.50 per consultation and for medication costs, SG$30.95 per vaccine, 100 days lost per outbreak (See Fig. 1), and a 5 % proportion of the vaccinated side effects requiring 1 day of medical leave. These values varied from 50 % to 150 % of their original values, and their effects on cost savings are evaluated *ceteris paribus* (See Table 2).

**Fig. 2:**
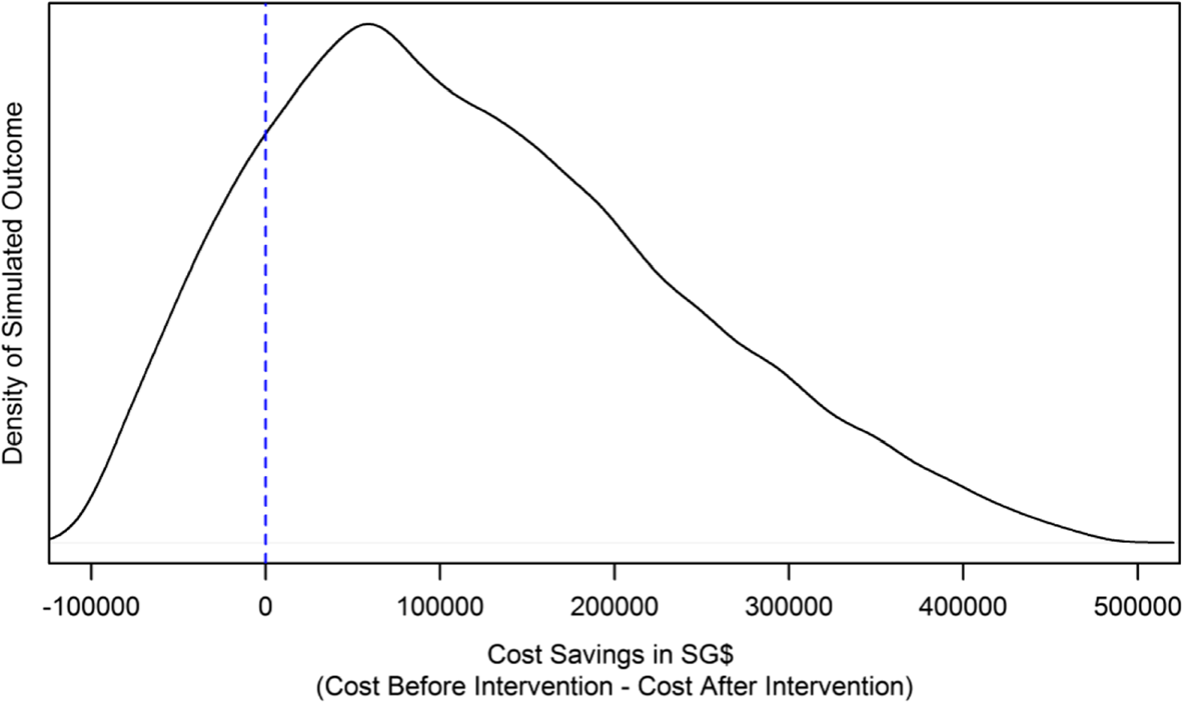
The Density of the simulated outcome

**Table 2:** Sensitivity analysis

Variable	Change in cost savings as a result of intervention Positive value favors intervention	Sensitivity
Income at year 2007	+$24.12 per $1 increment of income	Higher income means more valuable man-days. With more than 1600 MD savings per year, each dollar increase is a significant impact on the cost effectiveness of the intervention.
Days lost from outbreaks	+$5,704 per man day (from 100 MD to 101 MD)	Sensitive. Although there are few outbreaks, each outbreak represents a significant cost. Please refer to Fig. 1 to see how the vaccination program changed cost of handling the disease over time.
Estimated % with side effects	-$2600 per 0.1 % (from 5 % to 5.1 %)	Moderately sensitive. Due to the large number of vaccinations, increased percentage side effects will have an impact on the number of medical leave taken.
Consultation fee and medications	-$201.7 per $1 increment (from $34.5 to $35.5)	Changing consultation fee and medication cost barely affects the model.
Wage growth	+$406.3 per 0.1 % increase (from 6.09 % to 6.19 %)	Not sensitive. It takes a big change in wage growth to change the results.
Discount rate	+$3,593 (from 0.1025 % to 0.1125 % per month). 0.01 % per month is approximately equal to 16–17 basis points	Not sensitive. It requires a significant shift in 5Y risk-free return to change the result of the analysis.

## Results

The demographic information is shown in Table 3. For the duration of the study from December 2007 to December 2013, there were a total of 435 varicella cases in the at-risk population of 70,000 active servicemen during each time period. Their age range was between 18–49 years, with a mean age of approximately 22.7 years. As shown in Table 3, the characteristics of the varicella patients between the pre-intervention group and the post-intervention group appear to be largely the same. Three-quarters (76 %) of the population had a previous history of varicella infection or vaccination. No mortality cases or cases requiring hospitalization were noted during the 2 study periods. All figures were compounded to 2014 values using 5Y SGS rates.

**Table 3:** The number of varicella cases pre- and post-intervention

Demographics	Number of cases		*P*-value
	Pre-intervention (Dec 2007 - Nov 2010)	Post-intervention (Dec 2010 to Dec 2013)	
Sex			
Female	10 (3.2)	2 (1.6)	0.357
Male	301 (96.8)	122 (98.4)	0.357
Race			
Chinese	233 (74.9)	102 (82.3)	0.101
Malay	23 (7.4)	7 (5.6)	0.515
Indian	28 (9.0)	11 (8.9)	0.965
Others	27 (8.7)	4 (3.2)	0.046*
Job			
NSF	242 (77.8)	87 (70.2)	0.093
Non-NSF	69 (22.2)	37 (29.8)	0.093
Age			
<20	71 (22.8)	13 (10.5)	0.003*
20-22	172 (55.3)	70 (56.5)	0.828
23-25	25 (8.0)	11 (8.9)	0.776
26-30	16 (5.1)	12 (9.7)	0.082
31-35	15 (4.8)	13 (10.5)	0.030*
36+	12 (3.9)	5 (4.0)	0.933
Medical Leave (Workdays)			
0-2 days	90 (28.9)	38 (30.6)	0.724
1 week	115 (37.0)	46 (37.1)	0.981
>1 week	106 (34.1)	40 (32.3)	0.716

Numbers in brackets are the proportion of cases within that category, given as a percentage. The *P*-value is calculated as a 2-tailed *t*-test of proportions between categories. “Other Race” includes minority races in Singapore, such as Boyanese, Bugis, Burmese, Caucasian, Ceylonese, Eurasian, Javanese, Nepalese and Sikh. “NSF” refers to Full Time National Servicemen, who are male Singaporeans that are conscripted into the military under the Enlistment Act

There were a total of 311 varicella cases and 41 outbreaks from December 2007 to December 2010. The cost of treating and vaccinating against varicella was SG$74,687 (95 % CI: 72,397 - 76,929), with 6,128 work days lost (95 % CI: − 5,306 to 6,946), and the total costs from varicella, including the cost of days lost, the cost of outpatient treatment, the cost of vaccinations and the cost of outbreak management from December 2007 to December 2010 is SG$961,887 (95 % CI: − 471,356 to 1,449,954) (Table 4).

**Table 4:** Total costs before and after instituting vaccination for all pre-enlistees without a reported history of varicella infection or vaccination since 10 December 2010

Periods	Dec 1, 2007 – Dec 10, 2010	Dec 11, 2010 – Dec 20, 2013
Number of varicella cases	311	124
Total cost of days lost (SG$) (a)	$287,848 (116,862-395,768)	$126,007 (51,157-173,250)
Number of outbreaks	41	3
Number of outbreaks per 10000	5.86	0.429
Total cost of outbreak management (SG$) (b)	585,694 (272,200 - 962,630)	48,352 (22,472 – 79,471)
Total number of work days lost (from both sickness and outbreaks)	6,128 (5,306 - 6,946)	1,083 (1,023 - 1,143)
Total number of work days lost (per 1000 servicemen) (from both sickness and outbreaks)	87.5 (75.8 - 99.2)	15.5 (14.6 - 16.3)
Total cost of outpatient consultations and medications (SG$) (c)	$11,360 (9,066 – 13,600)	$4,380 (3,497 – 5,245)
Number of vaccines administered	1,935	16,750
Cost of vaccinations given (SG$) (d)	$63,331.25	$528,362.94
Cost of side effects of vaccines (5 % × total number given vaccines × cost of 1 lost day) (SG$) (e)	$13,658 (5,545-18,779)	$136,069 (55,242-187,084)
Cost of vaccines given + side effects (SG$) (d) + (e)	$76,990 (68,880-82,110)	$664,400 (583,600-715,400)
Total costs excluding work days lost (SG$) (c) + (d) + (e)	$88,340 (82,290-95,630)	$668,800 (588,800-720,400)
Total costs including work days lost (SG$) (a) + (b) + (c) + (d) + (e)	$961,887 (471,356 – 1,449,954)	$843,171 (662,409 – 971,688)

There were a total of 124 varicella cases and 3 outbreaks from December 2010 to December 2013. The cost of treating and vaccinating against varicella is SG$532,743 (95 % CI: 531,860 - 533,608) with 1,083 work days lost (95 % CI: 1,023 - 1,143), and the total costs from varicella, including the cost of days lost, the cost of outpatient treatment, the cost of vaccinations and the cost of outbreak management from December 2010 to December 2013 is SG$843,171 (95 % CI: 662,409 - 971,688).

This means that vaccinating all SAF servicemen upon enlistment would save 5,045 work days (95 % CI: − 4,283 TO 5,803) or 72.0 work days per 1000 (95 % CI: 61.2 - 82.9). To achieve work day savings, SG$458,100 (95 % CI: 456,700 - 459,500) or SG$6,544 per 1000 (95 % CI: 6,524 - 6,564) worth of vaccines and other medical costs will need to be invested. This works out to SG$91.5 (95 % CI: 78.7 - 107.3) per work day saved and a reduction of 187 (2.7 per 1000) varicella cases and 38 (5.43 per 10000) outbreaks. Taking into account the cost of work days lost over a three year period, compared with the previous regime of vaccinations only for selected individuals, the total savings is SG$118,700 (95 % CI: −191,100 - 478,300) or SG$1,695 per 1000 (95 % CI: −2,730 - 6,834) (Fig. 2).

The model is most sensitive to the estimated income and the estimated days lost from outbreaks. The intervention would be cost effective (from a purely monetary point of view) when the value of a working month exceeds SG$1,900. The model is also sensitive to the cost of vaccines, as this makes up most of the cost of the intervention (Table 2).

## Discussion

We have shown that the vaccination of servicemen determined to be without prior varicella vaccination or infection could lead to total cost savings due to reduced varicella infections and outbreaks.

Our findings are consistent with previous studies in militaries showing that the vaccination of servicemen susceptible to varicella reduces the incidence of chickenpox [18]. This is especially important because the communal military environments place personnel, particularly recruits, at a higher risk of varicella infection [19]. For example, studies found that the vaccination of newly enlisted sero-negative US military recruits reduced the overall incidence by more than 80 % and was also the most cost-effective decision [3, 4]. Furthermore, among US active-duty personnel, the adjusted risk of hospitalization fell more than two-fold from 1987–1988 to 1993–1994, possibly due to the introduction of varicella vaccination [5]. Our study noted a 60 % reduction in incidence (from 311 to 124 cases); however, as there were no hospitalization cases in both time periods, and thus the hospitalization incidence could not be compared.

An Israeli study of secular trends of chickenpox among the military population in Israel in relation to the introduction of varicella zoster vaccine 1979–2010 suggests that the rates of chickenpox in the military population have significantly declined since the introduction of the vaccine into the civilian population in Israel and have almost disappeared completely since 2008, when the vaccine was included in the state-funded routine childhood immunization schedule; however, this study did not include an economic evaluation of the vaccination program [6]. The authors further concluded that these findings underscore the need for a strong surveillance system and will aid in determining vaccination policies. Other militaries or similar organizations that require close contact between its members (like schools and manufacturing factories) could consider varicella vaccinations in their own settings, but further studies are crucial for determining local economic effectiveness. By comparison, our study was able to determine the economic effectiveness in the setting of the Singapore military.

The effect of varicella vaccination on herpes zoster (HZ) is unclear at present [20, 21]. Based on the hypothesis that exposure to varicella may boost immunity to latent VZV, the vaccination-associated decrease in varicella disease has led some to suggest that the incidence of HZ might increase. A US study mentioned that a vaccination-associated decrease in varicella disease did not result in an increase in the incidence of HZ but did not show a significant decrease either [22]. As costs associated with zoster have not been factored into our study, it is possible that our cost effectiveness results may be affected by the incorporation of herpes zoster data. However, this would require the follow up of SAF personnel many years into the future.

One of our assumptions is that the value of one lost day of work used was the median gross monthly income from work of full-time employed residents for 2012, rather than the salary estimates of SAF personnel. This is because the salary allowance for enlisted SAF servicemen is meant as an appreciation and is not a true reflection of the economic value of their contributions to Singapore’s defense. Additionally, the income of 21-year-old male Singaporeans was not used because most 21-year-old Singaporean males would either be enlisted in the SAF or pursuing full-time tertiary studies and therefore the reported “salary” for this age group is incorrect.

In the context of the Singapore Military, due to the paramount need to maintain Singapore’s defense capabilities, there must always be a pool of trained and healthy servicemen that are readily available at all times. Thus, even if the varicella vaccination regimen instituted after December 2010 was not cost saving, it is likely that Singapore would invest in such a program, as varicella could severely affect a military unit should a percentage of servicemen fall ill and have to be isolated. This is also because sufficient military defense ensures that the nation of Singapore remains secure, allowing economic and social progress to take place [23].

The cost of a varicella outbreak is estimated to be 100 man-days. Before the universal vaccination regime, when the bulk of the outbreaks took place, approximately 30 servicemen within a company of 100 soldiers would require vaccination, as, based on a prior serological study, they do not have antibodies against varicella [5]. Soldiers within the same company come into close contact with each other daily and are most likely to spread infectious diseases to each other. This means that approximately 50 man-days would be spent by these susceptible servicemen having to take a day off in their training to take turns receiving their vaccinations at the medical center, by their superiors required to manage the vaccination exercise and by the medical center staff ordering, delivering and administering the vaccinations to these servicemen. Additionally, it takes approximately 25 man-days of administrative measures such as activating the Preventive Medicine Unit staff and camp medical officers for contact tracing, meetings and writing reports. Another 25 man-days would be used by the Unit’s officers for daily screening of contact cases (the whole company) until the declared end of the outbreak (i.e., no cases after 2 incubation periods).

### Limitations

There is a possibility of the under-reporting of varicella cases, especially because varicella cases were diagnosed through symptom assessment only (sub-clinical or atypical cases may not be picked up), which could lead to the under-reporting of varicella cases in the vaccinated cohort. Additionally, under-reporting could occur because some affected servicemen were treated by doctors external to the military. However, all medical certificates from external medical practitioners beyond 3 days have to be endorsed by an SAF Medical Officer, who would review and enter the case into the electronic medical records system. Therefore, it is assumed that all servicemen who had symptomatic varicella infection (which lasts an average of 7 days) were seen by their SAF Unit Medical Officer.

The main weakness of a pretest-posttest design is the lack of a concurrent comparison group. For instance, the observed reduction in varicella incidence post-intervention may be attributed to external events such as increased uptake of the varicella vaccination in the general population during the post-intervention period. However, given that there had not been any major national effort promoting varicella vaccination during the study period and that chickenpox was already a well-known disease to Singaporeans because it had been endemic for decades, there is little reason to suspect that the varicella vaccination rate had changed profoundly over these 2 time periods. In addition, the size and socio-demographic characteristics of both groups is equivalent as the same number of personnel would be recruited to replace those who have completed their service.

Another limitation is the non-generalizability of this study to non-military settings. This is because the age distribution of the study population is predominantly young male enlistees, who have a different exposure and risk profile compared to general or pediatric populations. Moreover, the semi-closed communal environment that recruits train in facilitates the spread of disease (and resultant outbreaks), unlike the open environments of the general population. However, our results have high internal validity in the operational research setting of the Singapore Military.

We also note that the study does not take into account the societal value of health; thus, further studies are needed (e.g., cost-effectiveness and cost-utility analyses). This is because the inclusion of the macro-economic impact, i.e., the impact of a large outbreak or the impact of herd immunity on the performance, structure, behavior, and decision-making of the Singapore society and economy as a whole, rather than just within the SAF's context, would lead to a greater degree of favoring of interventions such as vaccinations. Additionally, increasing herd immunity from vaccination during National Service would reduce the incidence of varicella in the general Singaporean population.

## Conclusion

In conclusion, the current program of vaccinating all servicemen determined to have no prior varicella infection or vaccination should continue, as it leads to cost savings and a reduction in clinical disease when compared to the earlier vaccination strategy. Moving forward, further cost analyses could be conducted for other illnesses that are vaccine-preventable and could cause significant morbidity in the military.

## Abbreviations

GPs: general practitioners
HZ: herpes zoster
MOs: medical officers
SAF: Singapore armed forces
VZV: varicella zoster virus

## References

[1] Wood MJ (2000). History of varicella zoster virus. Herpes.

[2] Kaneshiro, N. Chickenpox. In: ADAM Inc. MedlinePlus - Trusted Health Information for You. U.S. National Library of Medicine. 2013. https://www.nlm.nih.gov/medlineplus/ency/article/001592.htm. Accessed 30 Aug 2014

[3] Ryan MA, Smith TC, Honner WK, Gray GC (2003). Varicella susceptibility and vaccine use among young adults enlisting in the United States Navy. J Med Virol.

[4] Jerant AF, DeGaetano JS, Epperly TD, Hannapel AC, Miller DR, Lloyd AJ (1998). Varicella susceptibility and vaccination strategies in young adults. J Am Board Fam Pract.

[5] Herrin VE, Gray GC (1996). Decreasing rates of hospitalization for varicella among young adults. J Infect Dis.

[6] Mimouni D, Levine H, Tzurel Ferber A, Rajuan-Galor I, Huerta-Hartal M (2013). Secular trends of chickenpox among military population in Israel in relation to introduction of varicella zoster vaccine 1979–2010. Hum Vaccin Immunother.

[7] Varivax Merck & Co. In: FDA U.S. Food and Drug Administration, Vaccines, Blood & Biologics. 2010. http://www.fda.gov/biologicsbloodvaccines/vaccines/approvedproducts/ucm200582.htm. Accessed 30 Aug 2014.

[8] Jean-Jasmin LM, Lynette SP, Stefan M, Kai CS, Chew FT, Wah LB (2004). Economic burden of varicella in Singapore--a cost benefit estimate of implementation of a routine varicella vaccination. Southeast Asian J Trop Med Public Health.

[9] American Academy of Pediatrics (2000). Committee on Infectious Diseases. Varicella vaccine update. Pediatrics.

[10] WEBMD. Chickenpox (Varicella) Vaccine, 2014. Available at: http://www.webmd.com/children/vaccines/chickenpox-varicella-vaccine. Accessed 30 August 14.

[11] Goldman GS (2005). Universal varicella vaccination: efficacy trends and effect on herpes zoster. Int J Toxicol.

[12] Kriner P, Lopez K, Leung J, Harpaz R, Bialek SR, Centers for Disease Control and Prevention (CDC) (2014). Notes from the Field: varicella-associated death of a vaccinated child with leukemia - California, 2012. MMWR Morb Mortal Wkly Rep.

[13] Dashraath P, Ong ES, Lee VJ (2007). Seroepidemiology of varicella and the reliability of a self-reported history of varicella infection in Singapore military recruits. Ann Acad Med Singapore.

[14] RStudio Team. RStudio: Integrated Development for R. In: RStudio, Inc., Boston. 2015. http://www.rstudio.com/. Accessed 30 Aug 2014

[15] Lee VJ, Tok MY, Chow VT, Phua KH, Ooi EE, Tambyah PA (2009). Economic analysis of pandemic influenza vaccination strategies in Singapore. PLoS One.

[16] Ministry Of Manpower, Comprehensive Labour Force Survey, 2013. http://stats.mom.gov.sg/Pages/Home.aspx. Accessed 28 Feb 2014.

[17] Wise RP, Salive ME, Braun MM, Mootrey GT, Seward JF, Rider LG (2000). Postlicensure safety surveillance for varicella vaccine. JAMA.

[18] Gray GC, Palinkas LA, Kelley PW (1990). Increasing incidence of varicella hospitalizations in United States Army and Navy personnel: are today's teenagers more susceptible? Should recruits be vaccinated?. Pediatrics.

[19] Longfield JN, Winn RE, Gibson RL, Juchau SV, Hoffman PV (1990). Varicella outbreaks in Army recruits from Puerto Rico. Varicella susceptibility in a population from the tropics. Arch Intern Med.

[20] Brisson M, Edmunds WJ (2003). Varicella vaccination in England and Wales: cost-utility analysis. Arch Dis Child.

[21] Brisson M, Gay NJ, Edmunds WJ, Andrews NJ (2002). Exposure to varicella boosts immunity to herpes-zoster: implications for mass vaccination against chickenpox. Vaccine.

[22] Jumaan AO, Yu O, Jackson LA, Bohlke K, Galil K, Seward JF (2005). Incidence of herpes zoster, before and after varicella-vaccination-associated decreases in the incidence of varicella, 1992–2002. J Infect Dis.

[23] Government Of Singapore. Total Defence. 2013. http://www.mindef.gov.sg/imindef/mindef_websites/topics/totaldefence/home.html. Accessed 30 August 14.

